# *Scrophularia arguta*, a widespread annual plant in the Canary Islands: a single recent colonization event or a more complex phylogeographic pattern?

**DOI:** 10.1002/ece3.2109

**Published:** 2016-05-26

**Authors:** Francisco Javier Valtueña, Josefa López, Juan Álvarez, Tomás Rodríguez‐Riaño, Ana Ortega‐Olivencia

**Affiliations:** ^1^ Área de BotánicaFacultad de CienciasUniversidad de Extremadura06006BadajozSpain

**Keywords:** Cryptic species, evolutionary stasis, island colonization, Macaronesia, molecular dating, phylogeography

## Abstract

Many studies have addressed evolution and phylogeography of plant taxa in oceanic islands, but have primarily focused on endemics because of the assumption that in widespread taxa the absence of morphological differentiation between island and mainland populations is due to recent colonization. In this paper, we studied the phylogeography of *Scrophularia arguta*, a widespread annual species, in an attempt to determine the number and spatiotemporal origins of dispersal events to Canary Islands. Four different regions, ITS and ETS from nDNA and *psbA‐trnH* and *psbJ‐petA* from cpDNA, were used to date divergence events within *S. arguta* lineages and determine the phylogenetic relationships among populations. A haplotype network was obtained to elucidate the phylogenetic relationships among haplotypes. Our results support an ancient origin of *S. arguta* (Miocene) with expansion and genetic differentiation in the Pliocene coinciding with the aridification of northern Africa and the formation of the Mediterranean climate. Indeed, results indicate for Canary Islands three different events of colonization, including two ancient events that probably happened in the Pliocene and have originated the genetically most divergent populations into this species and, interestingly, a recent third event of colonization of Gran Canaria from mainland instead from the closest islands (Tenerife or Fuerteventura). In spite of the great genetic divergence among populations, it has not implied any morphological variation. Our work highlights the importance of nonendemic species to the genetic richness and conservation of island flora and the significance of the island populations of widespread taxa in the global biodiversity.

## Introduction

Oceanic islands are considered “living laboratories” to study evolutionary processes and provide ideal systems to investigate historical colonization and evolutionary patterns in plants (Emerson 2002; Whittaker and Fernández‐Palacios 2007). The importance of oceanic islands in evolution studies is due to three main features: (1) They usually have a volcanic origin, (2) they initially do not have any terrestrial life, and (3) they currently have a high percentage of endemic taxa. Most studies on oceanic islands have been carried out in three archipelagos: Hawaii and Galapagos in the Pacific Ocean and Canary in the Atlantic Ocean.

The presence of a species in an oceanic island implies two main processes, dispersal and establishment. Dispersal to oceanic islands has been considered an infrequent event stochastic in nature (Heleno and Vargas 2015) which depends mainly on the distance to mainland or to other islands (Nathan 2006). Species establishment mainly depends on ecological factors and reproductive traits, so the species with wide ecological plasticity and self‐compatibility have more probabilities to establish after dispersal (Baker 1955; Chamorro et al. 2012). After taxa establishment, island populations often evolve differently to mainland populations due to distinct evolutionary pressures resulting in new morphologically different species or originating cryptic species in which the genetic differentiation has not resulted in phenotypic variation (morphological stasis) (Swenson et al. 2015). In the last years, some studies have shown the importance of cryptic species in the biodiversity of certain regions, which would be undervalued when only morphological species are considered (Adams et al. 2014 and references therein).

Most of the studies in oceanic islands have been focused in endemic species, where genetic differentiation with respect to their sister species or clades has been accompanied by phenotypic differentiation, but only a few studies have been carried out in widespread species. The presence of widespread species in oceanic islands together with the absence of speciation processes in such species is mainly explained by a relative recent colonization, so that has not gone long enough isolation and divergence to originate a new species. Likewise, it is generally considered that island populations have less genetic diversity (but see García‐Verdugo et al. 2015) due to founder effect (populations proceed from a few individuals), and by the absence of genetic differentiation they are not important for the global genetic richness of the species. Nevertheless, if the colonization event was ancient and island populations have been isolated, it could be possible to find that these populations are genetically different from mainland populations and they could become cryptic species. Other general hypothesis to explain the presence of widespread species in oceanic islands is that they were originated there and later colonized mainland (v.g., the genus *Aeonium* distributed by Macaronesia and Africa; Mes et al. 1996).

The Canary Archipelago is composed of seven main islands located in Macaronesia, a floristic region that also includes the Cape Verde, Selvagems, Madeira, and Azores archipelagos. In Macaronesia, most studies have been conducted in the Canary Islands because (1) they are the closest islands to the mainland (at present 90 km between Fuerteventura and northwest Africa), (2) they formed over a wide range of time (between 23 Ma of Fuerteventura and 1.1 Ma of El Hierro; Carracedo et al. 2002; van den Bogaard 2013), (3) they show a high percentage of endemism (45%, Caujapé‐Castells et al. 2010), and (4) they cover a wide range of habitats because of substantial elevation, soil, and annual rainfall (Hobohm 2000).

In recent years, many studies have focused on patterns of Canary Island colonization to determine the number of colonization events, mainland origin of these events, and the time frame in which they occurred (references in Silvertown 2004; Carine 2005; Díaz‐Pérez et al. 2008; among other). From these studies, several general patterns have emerged. Most Canarian genera reached the islands after one or a few colonization events (Silvertown 2004; Carine 2005). Although we know that dispersal from the mainland to islands should be considered uncommon, and, in many cases, due to unpredictable events (Heleno and Vargas 2015), an important proportion of Canarian founder taxa have long distance dispersal (LDD) adaptations (Bramwell 1985; Vargas 2007), so adaptations to LDD look to favor the likelihood of establishment. Furthermore, after island colonization and because of ecological niche availability, many taxa undergo speciation or evolutionary radiation processes (e.g., references in Carine 2005; Lledó et al. 2005; Kim et al. 2008; García‐Maroto et al. 2009), occupying these niches and decreasing the probability of establishment of similar or related taxa by competition (niche preemption hypothesis, Silvertown 2004). The most common pattern of Canary Island colonization follows the “stepping‐stone” model from east to west, which involves an initial colonization of the islands closest to the mainland and the sequential colonization of the western islands by dispersal from the nearest eastern island (Juan et al. 2000; Francisco‐Ortega et al. 2001, 2002; Allan et al. 2004; Cowie and Holland 2006; Dlugosch and Parker 2007; Ortega‐Olivencia and Catalán 2009).

The above patterns are primarily observed in Canarian or Macaronesian endemic taxa, because very few studies have focused on Canarian species with wide distribution, with the exception of two recent studies on shrubby species, *Erica arborea* (Désamore et al. 2011), and *Cistus monspeliensis* (Fernández‐Mazuecos and Vargas 2011a). *E. arborea*, a circum‐Mediterranean species, diverged in the Tertiary and has a complex phylogeographic history involving two different colonizations of Macaronesia, one ancient Madeira colonization and a more recent colonization of the Canary Islands and Madeira. The western Mediterranean *C. monspeliensis* is a species that more recently reached the Canary Islands in a single event dated c. 0.9 Ma. However, its colonization pattern within the islands is complex and differs from the stepping‐stone model, involving several dispersal events to some islands and from various other islands.

This study focuses in *Scrophularia arguta* Sol. in Aiton (Scrophulariaceae). The genus *Scrophularia* includes about 270 species (Ortega‐Olivencia and Devesa 1993), most of which are herbaceous perennials and suffruticose that have a primarily Holarctic distribution. For this genus, a strong affinity between the western Mediterranean region and Macaronesia has been suggested (Dalgaard 1979; Ortega‐Olivencia and Devesa 1993), and this was recently confirmed using molecular markers (Scheunert and Heubl 2014; Navarro‐Pérez et al. 2015). Eleven species and subspecies inhabit Macaronesia, of which eight are endemic (*S. hirta* Lowe, *S. racemosa* Lowe, *S. lowei* Dalgaard to Madeira; *S. calliantha* Webb & Berthel*, S. glabrata* Aiton, and three subspecies of *S. smithii* Hornem. to the Canary Islands) and three have a greater range (*S. auriculata* L. and *S. scorodonia* L. inhabit west and southwest Europe plus Morocco, and *S. arguta* is distributed from northwestern Africa to the Arabian Peninsula and Horn of Africa, with some isolated populations in the Iberian Peninsula; Ortega‐Olivencia 2009).

The colonization of Macaronesia by *Scrophularia* involved at least five different events (Navarro‐Pérez et al. 2015), two of which occurred in the Canary Islands. These events affected two different clades, one including all of the Canarian endemic taxa (plus *S. hirta*) and the other constituted by *S. arguta* and *S. lowei* (Scheunert and Heubl 2014; Navarro‐Pérez et al. 2015). Scheunert and Heubl (2014) analyzed one individual of *S. lowei* and three of *S. arguta* (one from Morocco and two from the Canary Islands), constituting a monophyletic clade, with *S. lowei* located at the base of this clade. Navarro‐Pérez et al. (2015), using only one individual from each species, dated the divergence to the Pleistocene–Pliocene transition. Consequently, the presence of *S. arguta*, one of the few annual *Scrophularia* species (A. Ortega‐Olivencia, pers. obs.), in the Canary Islands should be explained by a single recent colonization event. However, because of the few analyzed individuals and lack of a detailed study on this species, the possibility of a more complex colonization pattern cannot be ruled out.

Our main objectives in this study on *Scrophularia arguta* phylogeography are to: (1) determine the number of colonization events to the Canary Islands, (2) date the colonization events to find out whether colonization of these islands by this species was ancient or recent, and (3) determine the geographical origin of the colonization events, differentiating between northwest Africa and southwest Europe.

## Material and Methods

### Species studied

**Figure 1:**
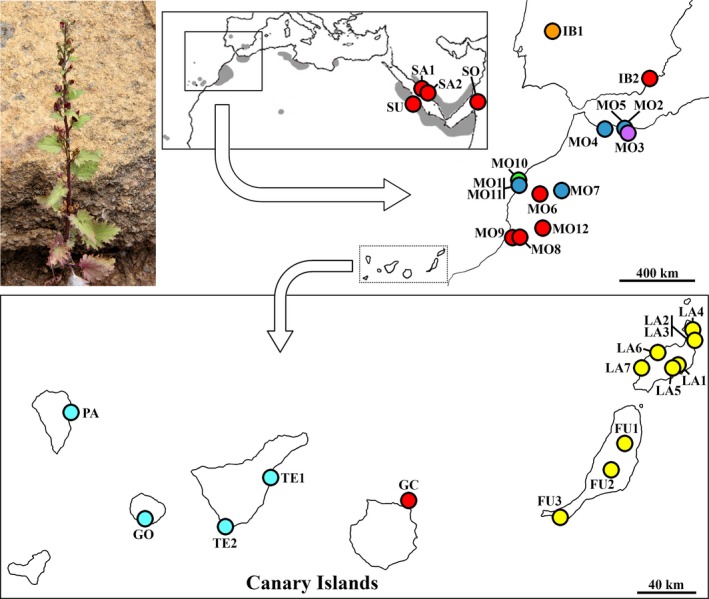
Habit and distribution map of *Scrophularia arguta* with location of studied populations. Population codes as in Table 1. Population colors indicate main haplotype as in Figure 4A.

*Scrophularia arguta* is an annual species distributed by northern and Horn of Africa, Arabia, Macaronesia (Canary Islands, Selvagem Islands, and Cape Verde), and with a few populations in southwest Europe (Fig. 1). Within *Scrophularia*,* S. arguta* reaches lowest latitudes (A. Ortega‐Olivencia, pers. obs.; Fig. 1) and is found beyond the Holarctic limit; additionally, it is the only amphicarpic species (plants with aerial chasmogamous as well as basal and/or underground cleistogamous flowers) and with a wide distribution in Macaronesia. *S. arguta* is present in all of the Canary Islands, with most of the known populations located in Lanzarote and Tenerife (Dalgaard 1979), although in general it is a rare species with very small and focalized populations. In the Canary Islands, it is usually found along the coast from sea level until 700 m, in dry rocky slopes, basaltic cliffs, and xeric lowlands.

### Sampling strategy, DNA extraction, and sequencing

We sampled a total of 33 *S. arguta* populations that covered most of its distribution (Fig. 1), including 15 populations from the Canary Islands (seven from Lanzarote, three from Fuerteventura, two from Tenerife, and one from each of the islands of La Palma, La Gomera, and Gran Canaria; in this last island, it is the unique known population) and 12 from Morocco (the closest area to the Canary Islands in which the species is found) (Table 1 and Fig. 1). In most populations, young leaves from at least two individuals were collected and kept in silica gel until their analysis. However, for some populations (GO, MO11, MO12, SA1, SA2, and SO), only one sample could be collected.

**Table 1:** Geographical origin of study material of *Scrophularia arguta* and cpDNA haplotype

Code	Location	Voucher	Collector	Haplotype
*Canary Islands*				
FU1	Fuerteventura, Tetir	UNEX 36128	JL & TRR	D_1_
FU2	Fuerteventura, Tiscamanita	UNEX 36129	JL & TRR	D_1_
FU3	Fuerteventura, Joros	UNEX 36130	JL & TRR	D_1_
GO	La Gomera, Barranco de Guarimiar	UNEX 36193	FJV & CM	G_2_
GC	Gran Canaria, La Isleta	UNEX 36192	FJV & CM	A_2_
LA1	Lanzarote, Tahiche	UNEX 36133	JL & TRR	D_2_
LA2	Lanzarote, Punta de las Mujeres	UNEX 36134	JL & TRR	D_2_
LA3	Lanzarote, Jameos del Agua	UNEX 36135	JL & TRR	D_3_
LA4	Lanzarote, Orzola	UNEX 36136	JL & TRR	D_1_
LA5	Lanzarote, San Bartolomé	UNEX 36137	JL & TRR	D_1_
LA6	Lanzarote, Tinajo	UNEX 36138	JL & TRR	D_1_
LA7	Lanzarote, El Golfo	UNEX 36139	JL & TRR	D_3_
PA	La Palma, Santa Cruz	UNEX 36194	FJV & CM	G_2_
TE1	Tenerife, Güimar	UNEX 36151	JL & TRR	G_1_
TE2	Tenerife, Pal‐Mar	UNEX 36152	JL & TRR	G_2_
*Iberian Peninsula*				
IB1	Spain, Cáceres, Santiago de Alcántara	UNEX 36131	AOO & FJV	C
IB2	Spain, Almería, Pulpí	UNEX 36132	AOO & FJV	A_6_
*Northwestern Africa*				
MO1	Morocco, Safi Cape	UNEX 36084	AOO & FJV	E_1_
MO2	Morocco, Zegangane	UNEX 36140	TRR, JL & FB	E_3_
MO3	Morocco, Hassi‐Berkane	UNEX 36141	TRR, JL & FB	F
MO4	Morocco, Had‐Rouadi	UNEX 36142	TRR, JL & FB	E_3_
MO5	Morocco, Beni‐Sidel	UNEX 36143	TRR, JL & FB	E_3_
MO6	Morocco, Sidi‐Bou‐Othmane	UNEX 36144	AOO & FJV	A_5_
MO7	Morocco, Oued El‐Abid Gorges	UNEX 36145	AOO & FJV	E_2_/E_1_
MO8	Morocco, Ouzaghar	UNEX 36146	AOO & FJV	A_2_
MO9	Morocco, Oued Assaka	UNEX 36147	AOO & FJV	A_2_
MO10	Morocco, Beddouza	UNEX 36148	AOO & FJV	B
MO11	Morocco, Safi	UNEX 36085	AOO & FJV	E_1_
MO12	Morocco, Jebel Agouti, Agadir Melloul	UNEX 36149	JJA	A_2_
*Northeastern Africa and Arabian Peninsula*				
SA1	Saudi Arabia, Jabal Hada	KSU 212279	AAG	A_4_
SA2	Saudi Arabia, Al‐Baha	KSU 17570	AHA	A_4_
SU	Sudan, Arkawit, Jebel Elsit	UNEX 36150	UB, SAC, PK	A_3_
SO	Yemen, Socotra Island, Fiheri Park	UNEX 36153	JJA	A_1_

AAG: A. Al‐Ghuraibi; AHA: A.H. Alfarhan; AOO: A. Ortega‐Olivencia; CM: C. Mayo; FB: F. Bueno; FJV: F.J. Valtueña; JJA: J.J. Aldasoro; JL: J. López; PK: P. Konig; SAC: S.A. Chaudhary; TRR: T. Rodríguez Riaño; UB: U. Bairele.

Genomic DNA was extracted using the Qiagen DNeasy Plant Mini Kit (Qiagen GmbH, Hilden, Germany), following the manufacturer's protocol. Two nuclear and two chloroplast (cp) regions were amplified and sequenced using the following primers: for the nuclear internal transcribed spacer (ITS), ITS5 and ITS4 (White et al. 1990); for the nuclear external transcribed spacer (ETS), 18S‐2L (Linder et al. 2000) and ETS‐Lar (Moore and Kadereit 2013); for the cp spacer *psb*J*‐pet*A, *psb*J and *pet*A (Shaw et al. 2007); and for the cp spacer *psb*A*‐trn*H, *psb*A‐F and *trn*H‐R (Sang et al. 1997). Polymerase chain reactions (PCRs) were performed in a volume of 25 *μ*l that contained 1× buffer, 0.2 mM dNTPs, 0.2 *μ*M of each primer, 4% DMSO, and one unit of *TaKaRa Ex Taq* polymerase (Takara Biotechnology, Otsu, Japan). The following amplification conditions were used: an initial denaturation for 1 min at 94°C; followed by 35 cycles of denaturation for 20 sec at 94°C, annealing for 30 sec at 52°C (for ITS, ETS, and *psb*J*‐pet*A) or 56°C (for *psb*A*‐trn*H), and elongation for 1 min at 72°C; and a final extension of 8 min at 72°C.

For all regions, both direct and reverse sequences were obtained. Sequencing was carried out by Service of Applied Techniques to Biosciences (Extremadura University, Badajoz, Spain). Sequences were manually checked and edited using Sequencher 4.10 (GeneCodes, Ann Arbor, MI, USA), and aligned by eye with MacClade 4.08 (Maddison and Maddison 2005). All sequences were submitted to GenBank (see Table S1).

### Dating of *Scrophularia arguta* lineages and phylogenetic analyses

To estimate the divergence and diversification times of *S. arguta* lineages, a Bayesian phylogenetic analysis of the ITS region was conducted with 135 sequences from *Scrophularia* taxa and three from other related Scrophulariaceae taxa (two *Verbascum* and one *Teedia* species) as outgroups. For *S. arguta*, we included 60 sequences, with two individuals per analyzed population, except for aforementioned populations (GO, MO11, MO12, SA1, SA2, and SO) where only one individual was used (see Table S1). Seventy‐seven sequences utilized in this analysis were obtained from GenBank, and 61 were obtained in this study (see Tables S1 and S2). The analysis was performed in Bayesian evolutionary analysis by Sampling Trees (BEAST) 1.8.2 (Drummond et al. 2012), with three calibration points obtained from a previous temporal calibration of a *ndh*F phylogeny (Navarro‐Pérez et al. 2013), which included minimum stem‐age constraints for Lamiales families and tribes based on five fossils following to Fernández‐Mazuecos and Vargas (2011b) and Vargas et al. (2014). The three calibration points implemented as normally distributed priors were (1) the split between *Teedia* and *Verbascum* plus *Scrophularia* (26.77 ± 4.27 Ma), (2) the split between *Verbascum* and *Scrophularia* (15.92 ± 3.29 Ma), and (3) the crown age of *Scrophularia* (10.20 ± 2.36 Ma).

The suitable substitution model was estimated using jModeltest 2.1.3 (Darriba et al. 2012). The GTR+G model was selected with gamma distribution modeled with six categories. *Verbascum* plus *Scrophularia* and *Scrophularia* were defined as monophyletic. A relaxed uncorrelated lognormal clock and a birth–death tree prior were used. Other priors were set to default values. Two Monte Carlo Markov chain (MCMC) (Drummond et al. 2002) analyses were initiated on a random starting tree with 20 million generations of each and a sample frequency of 1000. Satisfactory effective sample size (ESS) was reached after assessing convergence in TRACER 1.6 (Rambaut and Drummond 2007), as described in the BEAST manual (Drummond et al. 2007). The two resulting tree files were combined in LogCombiner 1.8.2 (Drummond et al. 2012) after discarding the first 10% of sampled generations as burn‐in. The maximum clade credibility (MCC) tree was summarized in TreeAnnotator 1.8.2 (Drummond et al. 2012) with a posterior probability (PP) limit of 0.90.

To infer the relationships among *S. arguta* populations, we used Bayesian inference (BI) and maximum likelihood (ML). Two datasets, one with the two nuclear regions (ITS and ETS) and other with the two nuclear regions and the chloroplast regions (*psb*A‐*trn*H and *psb*J‐*pet*A) concatenated, were examined using the incongruence length difference (ILD) test (Farris et al. 1994), implemented in PAUP version 4b.10 (Swofford 2002) with 100 replicates, 10 random addition sequences, tree–bisection–reconnection (TBR) branch swapping on best only, and MULTREES on. In both analyses, the results indicated that both partitions were similar as resulting from a random partition of the same size as the original partition (nuclear regions, *P* = 0.45; nuclear plus chloroplast regions, *P* = 0.74). Due to the congruence between the concatenated nuclear datasets (ITS and ETS regions) and the concatenated plastid plus nuclear dataset, both datasets were combined. The same *S. arguta* individuals from the first analysis were utilized, and one *S. megalantha* Rech. f. individual was used as the outgroup. Even though previous molecular studies (Scheunert and Heubl 2014; Navarro‐Pérez et al. 2015) indicate a sister relationship between *S. lowei* (endemic to Madeira) and *S. arguta*, another study found that *S. lowei* is not sister to but instead diverged from within an *S. arguta* lineage (F. J. Valtueña et al., unpubl. ms.). Consequently, it was not used as outgroup. We selected *S. megalantha* as the outgroup from a pool of species because it shows higher sequence identity with *S. arguta* in the analyzed sequences. Bayesian phylogenetic analyses were performed in BEAST 1.8.2 with the two concatenated nuclear datasets (ITS and ETS) combined with the two concatenated plastid datasets (*psb*A‐*trn*H and *psb*J‐*pet*A). This allowed us to infer relationships among *S. arguta* populations and divergence times among *S. arguta* lineages using two calibration points obtained from the first analysis, the *S. arguta* divergence age (3.28 ± 1.11 Ma) and crown age (10.45 ± 1.59 Ma). The substitution models selected using jModeltest 2.1.3 were GTR+G for the ITS+ETS dataset and the HKY+G for the cpDNA dataset. A coalescent constant size tree prior was used, and the remaining settings and calculation procedures were the same as in the previous dating analysis. A ML analysis was conducted in RAxML 8.1.11 (Stamatakis 2014) using a concatenated dataset of the four regions (ITS, ETS, *psb*A‐*trn*H, and *psb*J*‐pet*A) where two partitions were considered, one including the nuclear regions and another constituted by the plastid regions. This analysis was performed on XSEDE in the Cipres Portal (Miller et al. 2012), with automatic termination of bootstrapping by RAxML and the GTR+G substitution model.

Due to the usual different way of inheritance of the nDNA and cpDNA, both datasets were analyzed independently using BI and ML following the procedures indicated for the analysis of the combined datasets. Characteristics of analyzed datasets are detailed in Table S3.

### Haplotype networks and phylogeographic analysis of cpDNA

Haplotype networks were constructed to estimate the relationships among chloroplast haplotypes. Two different approaches were used to analyze the cpDNA matrix with 60 sequences (59 from *S. arguta*, which included all of the studied individuals in the previous analyses except for population FU3, where only one individual was used because it was impossible to amplify the *psb*A*‐trn*H sequence in the second one, as well as one from *S. megalantha*, which was used as the outgroup). Two approaches were used because the selected regions had several complex indels (between 5 and 152 bp) and included several polymorphic regions (poliA) that cannot be unambiguously codified. In the first approach, a dataset taking into account only the unambiguous complex indels (Table S3) coded as single characters was analyzed under statistical parsimony as implemented in the program TCS 1.21 (Clement et al. 2000), with a connection limit of 10 steps. For the second approach, a dataset with all of the unambiguous mutations (complex and single indels plus nucleotide substitutions), where the complex indels coded as single characters, was analyzed under statistical parsimony as implemented in the program TCS 1.21 (Clement et al. 2000), with a connection limit of 100 steps to include the outgroup.

The dispersal and diffusion of *S. arguta* were analyzed by Bayesian stochastic search variable selection (BSSVS, Lemey et al. 2009) of the discrete phylogeographic model implemented in BEAST 1.8.2. This analysis was conducted on the same individuals studied in the haplotype network, and six geographical areas were considered for *S. arguta*: Canary Islands, Iberian Peninsula, northwest Africa, East Africa, Arabian Peninsula, and Socotra Island. In the analyses, the coalescent model for the discrete geographical data was set with both symmetrical and asymmetrical substitution models, and the remaining settings and calculation procedures were the same as in the previous cpDNA analysis. Bayes factor (BF) analysis with SPREAD 1.0.7 (Bielejec et al. 2011) was used to identify well‐supported geographical state transitions with strong posterior support (BF ≥ 3).

## Results

### Dating of *Scrophularia arguta* lineages and phylogenetic analyses

**Figure 2:**
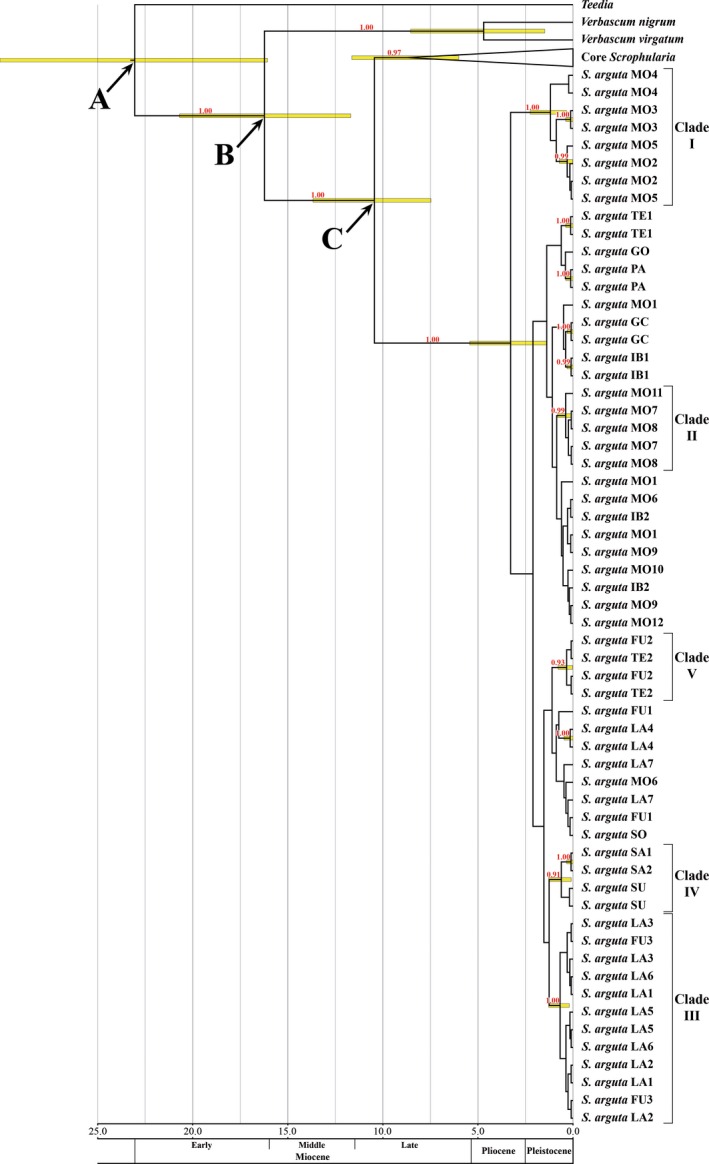
BEAST chronogram of *Scrophularia* based on ITS sequence variation. Posterior probabilities of clades are indicated above branches (only PP ≥ 0.90). The 95% posterior density distribution of node ages is shown in the node bars (only branches with a PP ≥ 0.90). The scale is in million years. Arrows indicate calibration points used in the analyses (A, 26.77 ± 4.27 Ma; B, 15.92 ± 3.29 Ma; C, 10.20 ± 2.36 Ma). Core *Scrophularia* includes all species of *Scrophularia* studied in the analysis except *S. arguta*; the complete tree is shown in Figure S1.

In the molecular dating using the marker ITS (Fig. 2, Fig. S1), *S. arguta* was highly supported (PP = 1.00) as the most basal lineage within the genus *Scrophularia*, placing its origin in the Miocene (10.45 Ma, 7.5–13.7 Ma, 95% highest posterior density, HPD, confidence interval), and the radiation of different lineages within this species during the Pliocene (3.28 Ma, 1.4–5.4 Ma HPD). In this analysis, there were three well‐supported clades (PP > 0.99) and two clades with poor support (PP between 0.90 and 0.95) involving more than one *S. arguta* population. Of the well‐supported clades, two consist of northwest African populations: Clade I is composed of the populations MO2, MO3, MO4, and MO5 (diversification 0.34–2.26 Ma), and clade II is composed of the populations MO7, MO8, and MO11 (0.07–0.81 Ma). Although clade I includes populations from the same area, clade II comprises individuals from geographically separated populations and has no supported relationship with other nearby populations (see Fig. 1). The third well‐supported clade (clade III) consists solely of Canarian populations, grouping together the majority of Lanzarote populations with one Fuerteventura population (0.20–1.28 Ma). Of the two poorly supported clades, clade IV includes the eastern populations of the species (Sudan and Saudi Arabia populations; 0.13–1.31 Ma), with the exception of the Socotra population (SO). Alternatively, clade V consists of a Tenerife population and a Fuerteventura population (0.03–0.80 Ma).

**Figure 3:**
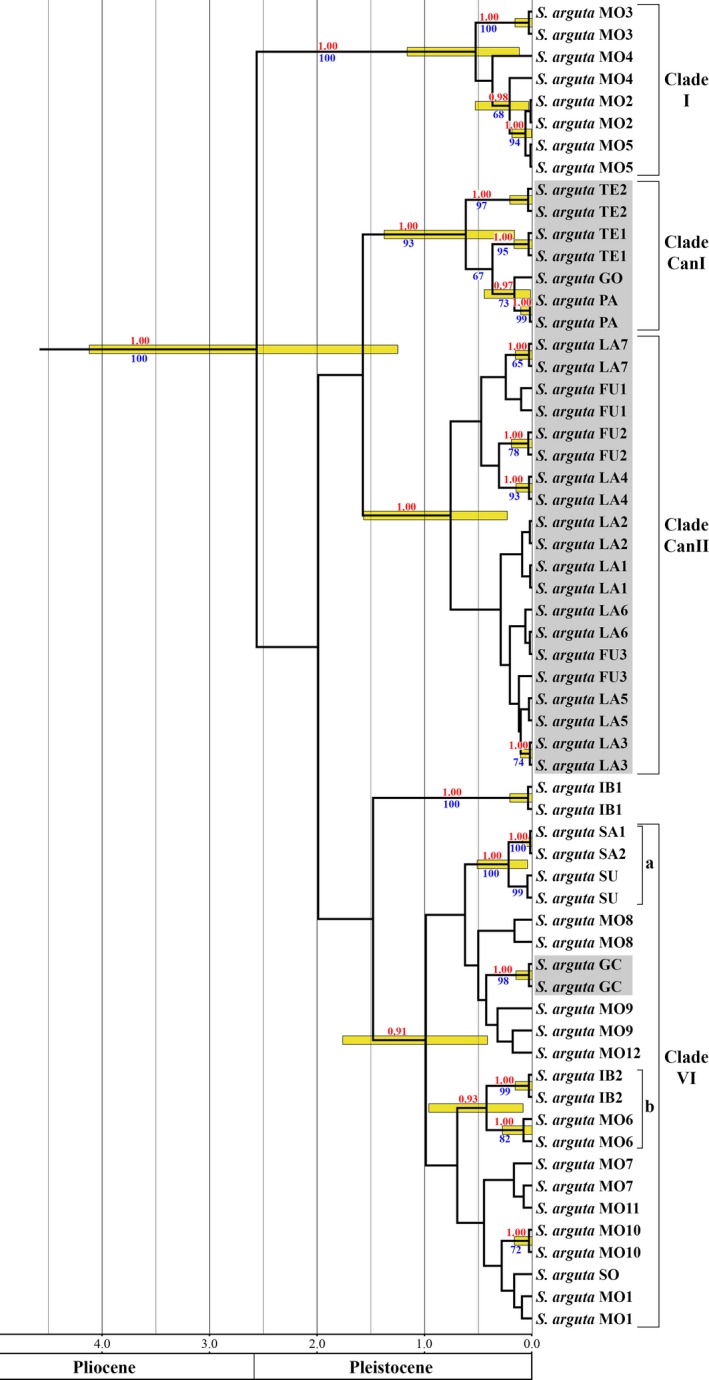
Chronogram of *Scrophularia arguta* based on the combined nDNA (ITS/ETS) and cpDNA (*psb*A*‐trn*H*/psb*J*‐pet*A) dataset obtained with BEAST. Values above branches are posterior probability (PP) values and under branches are maximum‐likelihood (ML) bootstrap (BS) values. Only PP ≥ 0.90 and ML BS ≥ 65 are shown. Gray background indicates the Canarian populations.

The analysis of the combined nuclear (ITS and ETS) and chloroplast (*psb*A‐*trn*H and *psb*J‐*pet*A) sequences focused only on *S. arguta* shows three well‐supported clades composed of individuals that belonged to more than one population (Fig. 3). Clade I (PP = 1) has the same composition that in the previous molecular dating analysis but differentiation is younger than in the previous analysis (0.12–1.16 Ma). The other two well‐supported clades are constituted only by Canarian populations: Clade CanI (PP = 1) groups the populations from Tenerife, La Gomera, and La Palma, whereas clade CanII (PP = 1) includes all the populations from Fuerteventura and Lanzarote. In both cases, the differentiation of these populations within each clade dates back to the Pleistocene (CanI: 0.16–1.38 Ma; CanII: 0.23–1.57 Ma). In clade CanI, population from La Gomera is sister to population from La Palma, whereas the relationships of the Tenerife populations are not solved. Finally, there is a poor supported clade (clade VI, PP = 0.91) constituted by populations from the entire range of the species which differentiated in the Pleistocene (0.42–1.76 Ma), and two subclades can be distinguished into it. Subclade VIa (PP = 1.00, differentiation: 0.04–0.51 Ma) corresponds to previous clade IV and is composed of the easternmost populations, except for the Socotra population; Subclade VIb is poorly supported (PP = 0.93) and groups one population from Iberian Peninsula and one population from northwest Africa (0.08–0.96 Ma). In addition, clade VI includes the Canarian population from Gran Canaria Island, although the relationships of this population with the rest of populations of the clade are not solved.

The results of ML analysis of the four concatenated regions with two partitions (nDNA and cpDNA) were similar to those found in the Bayesian analysis of these regions but with a poor resolution (Fig. 3). So, in this analysis, the clades VI and CanII were not supported. However, in clade CanI, populations from La Gomera and La Palma derived from Tenerife, although with poor support (BS = 67).

The analysis of the cpDNA produced similar results to the obtained in the previous combined analysis (nDNA plus cpDNA), but with the clade VI not supported (Fig. S2). Indeed, a clade grouping clade VIa plus populations from Gran Canaria and northwest Africa was well supported (Fig. S2). In the nDNA tree, the resolution was poorer and only clades I and VIa were supported with the same individuals that in the previous analysis of the combined nDNA and cpDNA (Fig. S3). However, the only incongruence in the topology obtained in this analysis was the grouping of one population from Tenerife and one population from Fuerteventura (TE2 and FU2, Fig. S3).

### Haplotype networks and phylogeographic analysis of cpDNA

**Figure 4:**
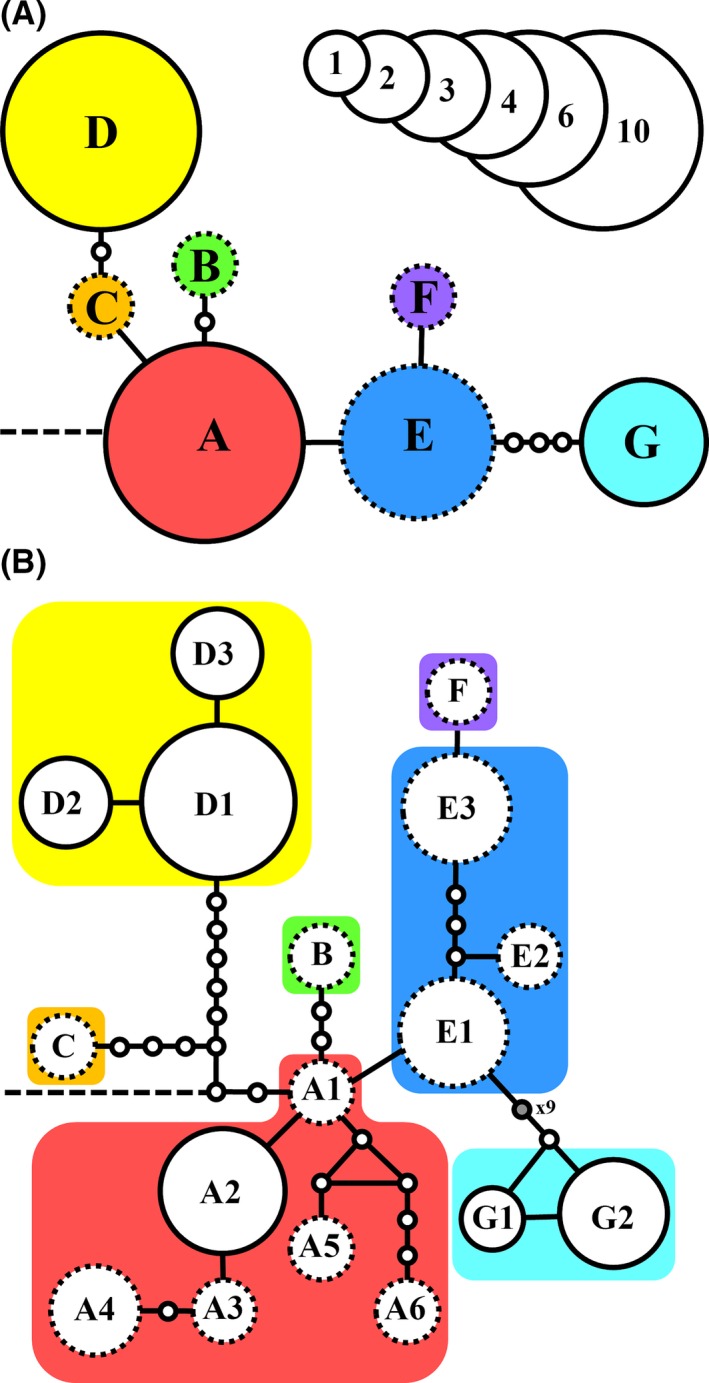
TCS statistical parsimony network of cpDNA haplotypes found in *Scrophularia arguta* for the matrix taking into account only complex gaps as mutational steps (A) and the matrix including all of the unambiguous mutations (B). In both analyses, gaps were codified as single mutations, the broken line indicates the connection to the outgroup (*S. megalantha*), and haplotypes including Canarian populations are indicated by a solid line at the edge. Small circles represent inferred mutational steps (open, one step; gray, more than one step, as indicated). The size of the haplotype symbol indicates the number of populations in which it has been found. Haplotype abbreviations are as in Table 1.

By considering only the complex indels, the analysis differentiates seven haplotypes with a more or less radial pattern within the lineage of *S. arguta* (Fig. 4A). The central position is occupied by haplotype A, which directly connects to the outgroup, and includes four northwest African populations, one population of southwestern Europe, all of the populations from the eastern distribution range (Sudan, Saudi Arabia, and Socotra Island) and one Canarian population (GC) (see Fig. 1). The other Canarian populations present haplotypes D and G, which are unique to the Canarian populations. Haplotype D is shared by all Fuerteventura and Lanzarote populations and differs by two mutational steps from haplotype C which is exclusive of one southwest European population (Fig. 1). Haplotype G, shared by populations from Tenerife, La Gomera, and La Palma, is four mutational steps from haplotype E, located only in northwestern Africa populations (Fig. 1).

When considering all unambiguous mutations, the haplotype network maintained the radial pattern (Fig. 4B). Haplotype A is composed of six different subtypes, most of which are found in a single population except haplotype A_2_ which is shared by three northwestern Africa populations and the Canarian population GC. The central position is occupied by haplotype A_1_ (exclusively found in Socotra Island and connected to the outgroup). The most divergent haplotypes (D and G) are found in Canarian populations. Haplotype D is composed of three subtypes, and haplotype G includes two subtypes (both present in Tenerife). The highest differentiation in the haplotype network happens between the haplotypes D_2_/D_3_ and G_1_/G_2_, differing between them in twenty‐one mutational steps.

In the phylogeographic reconstruction based on cpDNA, northwestern Africa is identified as the ancestral region for *S. arguta* (Fig. S4), with posterior probabilities of 0.67 and 0.86 for the symmetrical and asymmetrical models, respectively. In both analyses, three different colonization events of Canary Islands can be distinguished, all of them with northwestern Africa as the range of the ancestor with the highest probability (Fig. S4). The symmetrical model detected five significant connections (dispersal routes) among geographical regions versus seven connections by the asymmetrical model (Table S4). In both models, the only connection including the Canarian Islands implied the dispersal from and/or to Arabian Peninsula (Table S4).

## Discussion

### Origin and radiation of *Scrophularia arguta*

Molecular dating using the ITS region estimated that *S. arguta* diverged toward the middle–late Miocene (7.5–13.7 Ma) and is the basal lineage of *Scrophularia*. Navarro‐Pérez et al. (2013), using the ITS and *trn*L‐*trn*F regions, estimated differentiation of the *Scrophularia* lineages in the middle Miocene (6.06–14.89 My); in their analysis, they included a sequence of *S. arguta* and another of *S. lowei*. Both species were sister taxa and diverged in the Pleistocene, but these authors did not reveal the relationship of these species relative to the rest of the genus. These authors also did not determine which species were at the basal position in the phylogeny of the genus due to the existence of a polytomy in the base that included the *S. arguta* and *S. lowei* clade. Those results are consistent with ours if the basal position of *S. arguta* obtained in our analysis is correct, considering that *S. lowei* diverged from a *S. arguta* lineage and therefore is not a sister lineage (F. J. Valtueña et al., unpubl. ms.). A biogeographical study on the genus *Scrophularia* in Macaronesia using the ITS and three chloroplast regions also failed to clarify which species or clade is basal in the genus, but *S. arguta* is part of a clade whose base is unresolved and diverged toward the middle–late Miocene (Navarro‐Pérez et al. 2015). Therefore, previous studies do not contradict our result that *S. arguta* is an ancient species that originated in the Miocene, although they do not support its basal position within the genus.

Our molecular dating analysis using the ITS region estimated that differentiation of the current *S. arguta* lineages occurred c. 3.28 Ma ago (Pliocene, 1.38–5.43 Ma, 95% HPD). That period was characterized by the formation of the Mediterranean climate and aridification of North Africa (Suc 1984), East Africa, and the Arabian Peninsula (Cane and Molnar 2001). *S. arguta* is a species that inhabits xeric environments throughout its range, with preference for rocky terrains, cracks, crevices, rocky ledges and cliffs, xerophytic communities, as well as olive and palm groves, from sea level to 700 m (1100 m in Cape Verde) (Dalgaard 1979; Ortega‐Olivencia 2009, and A. Ortega‐Olivencia, pers. obs.). Therefore, it is feasible that Pliocene events facilitated its expansion by North Africa. The great time difference between the origin of *S. arguta* lineage (middle–late Miocene) and its diversification (Pliocene) could indicate that during this period this species presented a narrow distribution or a significant gene flow between its populations causing the homogenization of its haplotype and avoiding diversification; alternatively, previously to its diversification, this species could suffer an important bottleneck that wiped off any previous differentiation, surviving only the lineage that diversified later.

The radial pattern of haplotypes along with the lack of resolution in the phylogenetic trees, which is typical of radiation and demographic expansion processes (Szövényi et al. 2006), indicates that *S. arguta* differentiation probably occurred by rapid expansion in the Pliocene. The central position of the haplotype found on the island of Socotra would indicate that this expansion occurred from the east of its range to the west, where the most divergent haplotypes appear (haplotypes D and G, both specific to the Canary Islands). This pattern (rapid east–west expansion by North Africa) has also been observed in *Erica arborea* (Désamore et al. 2011). However, in this Mediterranean–Macaronesian shrub that reaches East Africa, colonization of West Africa and Macaronesia took place more recently (between 0.53 and 2.38 Ma). North Africa aridification did not favor the expansion of *E. arborea*, but caused the fragmentation of its range by extinction of North African populations; the wave of colonization of this territory coincided with the favorable periods of the Pleistocene (Désamore et al. 2011). On the contrary, the BSSVS analysis situated the ancestral distribution range of *S. arguta* in northwestern Africa, which implies that expansion occurred mainly from west to east. However, this analysis only considers cladogenetic evolution and together with support absence in the base of the tree and in the relationships between the main clades, this makes that this result cannot be considered suitable. For the same reason, the connections identified in this analysis cannot be considered as the real dispersal routes of this species across its distribution range.

A similar situation to *Erica* appears in *Myrtus communis*, a shrub with a similar distribution, which differentiated in two main lineages in the eastern and western of the Mediterranean region during the Late Miocene because of the Messinian salinity crisis (Migliore et al. 2012). Unlike *Erica*, both lineages of *Myrtus* rapidly expanded and differentiated during the Plio–Pleistocene throughout the Mediterranean basin (Migliore et al. 2012). In the past, the ecological optimum in both *Erica* and *Myrtus* would be located in the laurisilva, which would explain the different temporality of *S. arguta* expansion and differentiation. Although the latter species expanded and differentiated in the Pliocene as a result of accentuated aridity, both shrubby species suffered a reduction in range that led to genetic differentiation between eastern and western populations, and their subsequent expansion under favorable conditions (less xeric) during the late Pliocene and the Pleistocene. Nevertheless, the geographical origin of the expansion in our studied species should be interpreted with caution because of the lack of samples from the central part of North Africa, which could have been the central area from which the species spread. However, we do not know of any other plant species that originated from this area.

Previously, it had been indicated that taxa with Pan‐African disjunct distributions have these distributions because of dispersal and colonization events during favorable periods followed by divergence or differentiation in situ in certain refugia (Sanmartín et al. 2010; Alarcón et al. 2013). The differentiation in *S. arguta* is temporally similar to that found in a clade of *Campanula* subgr. *Azorina* (3.4 Ma; range 2.9–8.5 Ma) composed of three species (*C. hypocrateriformis, C. jacobaea*, and *C. balfourii*) distributed by Macaronesia, North Africa, and Socotra Island (Alarcón et al. 2013). Although this subgroup contains three different species, for *S. arguta*, with a very similar distribution, there were no speciation events or morphological differentiation, as shown by the fact that there has been no described infraspecific taxon worthy to be taken into account (A. Ortega‐Olivencia, pers. obs.). Furthermore, within *Scrophularia*, there is a clade that consists of four Macaronesian species (*S. calliantha*,* S. hirta*,* S. smithii,* and *S. glabrata*) whose speciation occurred during a similar time frame as the *S. arguta* differentiation (Navarro‐Pérez et al. 2015).

Consequently, it may be that the current *S. arguta* populations are maintaining in evolutionary stasis despite the clear genetic differentiation that exists between them. After LDD‐associated colonization events, it is thought that populations established in the new area show substantial divergence from the source populations (Ibrahim et al. 1996). This was observed in our study, with island populations showing greater divergence compared with the other populations. In addition, population differentiation would be enhanced by the characteristics of *S. arguta*, which is an annual species and therefore has a short life cycle and whose reproductive success is largely related to the production of cleistogamous flowers. It is known that there is often high genetic differentiation among populations in species that predominantly reproduce by selfing (Charlesworth and Charlesworth 1995; Hamrick and Godt 1996; Zhang et al. 2006; Voss et al. 2012).

### Colonization of the Canary Islands by *Scrophularia arguta*

Our results support that colonization of the Canary Islands occurred at least three times and, therefore, rejects the hypothesis of a single dispersal event and posterior geographical “stepping‐stone” pattern of colonization, because the island populations studied (1) are not monophyletic and (2) include the most divergent haplotypes. So, our results indicate that Canary Islands were colonized twice in ancient times originating the two most divergent haplotypes in the species (haplotypes D and G) and each event implying a different group of islands (haplotype D is located only in eastern islands whereas haplotype G is unique in western islands). In addition, there was a third colonization event which, in contrast to the previous two, was much more recent and involved only one island, Gran Canaria, located between the two groups of islands with the most divergent haplotypes.

From our knowledge, this study shows the greatest number of colonization events (three) of an oceanic archipelago by a plant species and involving two different temporal windows of colonization. A similar result was found in *E. arborea*, whose colonization of the Canary Islands involved two separate temporal events (Désamore et al. 2011).

For *S. arguta*, both the most divergent position of the exclusive Canarian lineages in the haplotype network and population divergence of these lineages (estimated in the Pleistocene) support the idea that the two most ancient colonization events of the Canary Islands occurred at a similar time and nearby to the differentiation age of the species, that is, before the onset of the Quaternary. These events also likely involved different areas from which there was dispersion to the Canary Islands, because the lineages do not share a common origin and the haplotypes that are closest to each lineage are not directly related. Furthermore, our results do not support the recolonization of the mainland from island populations (island‐to‐continent colonization hypothesis, Carine et al. 2004).

The colonization of western Canary Islands from the mainland was previously revealed for other species with widespread distributions, such as *Cistus monspeliensis* (Fernández‐Mazuecos and Vargas 2011a), being Tenerife the first island colonized. In this species, populations from Tenerife are closely related to those from Gran Canaria whereas in *S. arguta*, the only population studied (the unique known population so far) is related with northwestern Africa and not with any other Canarian populations. Unfortunately, there are few known populations in western Canary Islands, so the small number of populations collected and studied in these do not allow to explain the colonization pattern among these islands; however, the fact that in Tenerife both populations have different haplotypes and one of them is shared with the studied populations in La Gomera and La Palma could indicate that Tenerife was the first western island colonized and the events of colonization of the other western islands have been recent. It could be supported because Tenerife is the closest island with haplotype G to mainland. Furthermore, in the haplotype network, haplotype G is the most divergent and connected to haplotypes present in mid‐western and northern Morocco, which is a portion of the distribution that is the farthest from the populations studied in northwestern Africa with respect to the Canary Islands. However, if the colonization of western islands occurred in the Pliocene, there were a number of islands between Morocco and Tenerife that could have served as a bridge for the colonization of these islands (Fernández‐Palacios et al. 2011). This would be supported if the Selvagem Island *S. arguta* populations (located to the north of Tenerife and mid‐way between the Canary Islands and northern Morocco) were grouped with those of the western Canary Islands or there were intermediate haplotypes among western Canary Islands and northern Morocco populations. Another hypothesis to explain the greater similarity between the western Canary Islands and mid‐western as well as northern Morocco populations is that these populations were distributed further south at the time of the colonization of western Canary Islands and were subsequently replaced by populations with haplotype A.

The region of southwestern Morocco between Safi and Cap Dráa has great floristic similarities to the Canary Islands, and it has even been considered part of Macaronesia by some authors (Kim et al. 1996). However, subsequent studies reject this hypothesis and support that the similarities are because that region has been the primary source for plants that colonized the Canary Islands (Médail and Quézel 1999). This idea supports that this region is the origin of the Fuerteventura and Lanzarote populations in old times and the origin of the colonization of Gran Canaria Island in recent times. Similarly, it was previously proposed that there were two colonization events of the easternmost Canary Islands from the same region for the genus *Matthiola* (Jaén‐Molina et al. 2009). However, unlike *S. arguta*, the *Matthiola* colonization events involved species that form a monophyletic group and therefore do not correspond to two different lineages. Furthermore, in *Matthiola*, it was found that there was mainland recolonization from the islands, which we did not find with *S. arguta*. Surprisingly, the haplotype found in Gran Canaria Island is shared with some northwest African populations, and it is not related to the other Canarian haplotypes. This haplotype (A_2_) appears in the nearest studied Moroccan populations to Canary Islands, so they should be the origin of the Gran Canaria population by a recent LDD, due to the absence of differentiation in the island population. In any case, we cannot reject the possibility of an accidental human introduction in this island. In support of this is the fact that in the most recent list of wild vascular plants of the Canary Islands, about 27% of the species are considered introduced (Arechavaleta et al. 2010). However, *S. arguta* is considered native to Gran Canaria, and in addition, most introduced species in the Canary Islands are ornamental plants that escape from gardens (García Gallo et al. 2008).

Lanzarote and Fuerteventura share haplotype D and form a monophyletic group (clade CanII), which can be expected because these islands were a single land mass until recent times (Fernández‐Palacios et al. 2011). This clade diversified into three distinct haplotypes (D1–D3), whereas the other exclusive Canarian clade (CanI) comprises two subtypes of haplotype G. This low diversity in island populations, especially when compared with the northwest Africa populations where seven different haplotypes occur, could indicate that the two Canarian lineages have undergone a genetic bottleneck after colonization. Other studies indicate, however, similar or higher levels of genetic variation in widespread island taxa compared with continental relatives (reviewed in Stuessy et al. 2014). An example of the latter situation is *Cistus monspeliensis*, which have in Canary Islands greater variability than in Mediterranean populations and populations from the same Canarian islands have different haplotypes that are shared with other islands, indicating several colonization events from and to various islands (Fernández‐Mazuecos and Vargas 2011a). Nevertheless, in our study, we have not found any island where the two Canarian haplotypes co‐occur, which could support the niche preemption hypothesis (Silvertown 2004); thus, *S. arguta* establishment in an island prevented subsequent colonizations of the species, and the different haplotypes found in some islands are consequence of the diversification of the original colonizing haplotype. To confirm this, it should be necessary to extend the sampling in the western islands, where only one or two populations could be collected by island, despite an intense search.

The grouping of one population from Tenerife with other from Fuerteventura in the nDNA analysis, relationship that is not supported in the other analyses performed, could be explained by the different inheritance of the nuclear and chloroplast genomes (biparentally vs. maternally) in most of the angiosperms (Petit and Vendramin 2007), so, our results should imply the existence of gene flow between both areas. The main pollinators in this species are bees and hoverflies (T. Rodríguez‐Riaño, unpubl. data), and it looks very unlikely that these pollinators moved between both areas; therefore, the most likely hypothesis for our results would imply seed dispersal between islands. The absence of signals of this dispersal between islands could be due to the incapacity to establish new permanent populations in the islands (supporting the niche preemption hypothesis, Silvertown 2004) or to the existence of hybrid populations that have not been sampled in our study.

Finally, the great genetic differentiation between the two Canarian lineages has not resulted in phenotypic differentiation. However, it should be necessary to increase the number of studied nuclear markers to confirm that this differentiation found in chloroplast genome is extended to the complete species genome. If so, the *S. arguta* complex would be constituted at least by three cryptic species, two of them unique to the Canary Islands, and as it has been indicated for other organisms, both cryptic species do not co‐occur in the same island (Vodă et al. 2015).

## Data Accessibility

Genbank accession numbers of all DNA sequences are provided in Tables S1–S2.

## Conflict of Interest

None declared.

## Supporting information


**Table S1.** Material of *Scrophularia arguta* and outgroup taxon (*S. megalantha*) studied, including population code (as in Table 1), location and GenBank accession numbers for the DNA sequences analyzed.
**Table S2.** Taxa used in the ITS data set for dating the origin and diversification of *Scrophularia arguta*, including GenBank accession numbers (GBN).
**Table S3.** Characteristics of DNA sequence datasets and number of unambiguous indels used in the analysis of *Scrophularia arguta*.
**Table S4.** Bayes factor (BF) support for the significant connections (BF > 3) between geographical areas based on BSSVS analysis of cpDNA in *Scrophularia arguta* by using symmetrical and asymmetrical models.
**Figure S1.** BEAST chronogram of *Scrophularia* based on ITS sequence variation. Posterior probabilities of clades are indicated above branches (only PP ≥ 0.90).
**Figure S2.** BEAST chronogram of *Scrophularia arguta* based on two cpDNA sequences (*psb*A*‐trn*H*/psb*J*‐pet*A).
**Figure S3.** BEAST chronogram of *Scrophularia arguta* based on two nDNA sequences (ITS/ETS). Values above branches are posterior probability values (PP) and under branches are maximum‐likelihood (ML) bootstrap (BS) values.
**Figure S4.** Maximum clade credibility tree generated by BSSVS analysis of cpDNA in *Scrophularia arguta* considering symmetrical (a) and asymmetrical (b) models. Branches are colored according to highest probability inferred ancestral geographical range.
