# Supplementary material for: Scrophularia arguta, a widespread annual plant in the Canary Islands: a single recent colonization event or a more complex phylogeographic pattern?
Source: Ecol Evol. 2016 May 26;6(13):4258–73. doi: 10.1002/ece3.2109 (PMC4930978; doi:10.1002/ece3.2109)

**Supporting information**

***Scrophularia arguta*, a widespread annual plant in the Canary Islands: a single recent colonization event or a more complex phylogeographic pattern?**

Francisco J. Valtueña, Josefa López, Juan Álvarez, Tomás Rodríguez-Riaño and Ana Ortega-Olivencia

| **Table S1** Material of *Scrophularia arguta* and outgroup taxon (*S. megalantha*) studied, including population code (as in Table 1), location and GenBank accession numbers for the DNA sequences analyzed. | | | | | |
| --- | --- | --- | --- | --- | --- |
| Code | Location | ETS | ITS | *psb*A-*trn*H | *psb*J*-pet*A |
| *Canary Islands* | | | | | |
| FU1 | Fuerteventura, Tetir | KU945636  KU945637 | KU926629  KU926630 | KU945695  KU945696 | KU945756  KU945757 |
| FU2 | Fuerteventura, Tiscamanita | KU945638  KU945639 | KU926631  KU926632 | KU945697  KU945698 | KU945758  KU945759 |
| FU3 | Fuerteventura, Joros | -  - | KU926633  KU926634 | KU945699  - | KU945760  KU945761 |
| GO | La Gomera, Barranco de Guarimiar | KU945694 | KU926686 | KU945752 | KU945813 |
| GC | Gran Canaria, La Isleta | KU945690  KU945691 | KU926684  KU926685 | KU945750  KU945751 | KU945811  KU945812 |
| LA1 | Lanzarote, Tahiche | KU945644  KU945645 | KU926639  KU926640 | KU945704  KU945705 | KU945766  KU945767 |
| LA2 | Lanzarote, Punta de las Mujeres | KU945646  KU945647 | KU926641  KU926642 | KU945706  KU945707 | KU945768  KU945769 |
| LA3 | Lanzarote, Jameos del Agua | KU945648  KU945649 | KU926643  KU926644 | KU945708  KU945709 | KU945770  KU945771 |
| LA4 | Lanzarote, Orzola | KU945650  KU945651 | KU926645  KU926646 | KU945710  KU945711 | KU945772  KU945773 |
| LA5 | Lanzarote, San Bartolomé | KU945652  KU945653 | KU926647  KU926648 | KU945712  KU945713 | KU945774  KU945775 |
| LA6 | Lanzarote, Tinajo | KU945654  KU945655 | KU926649  KU926650 | KU945714  KU945715 | KU945776  KU945777 |
| LA7 | Lanzarote, El Golfo | KU945656  KU945657 | KU926651  KU926652 | KU945716  KU945717 | KU945778  KU945779 |
| PA | La Palma, Santa Cruz | KU945692  KU945693 | KU926687  KU926688 | KU945753  KU945754 | KU945814  KU945815 |
| TE1 | Tenerife, Güimar | KU945684  KU945685 | KU926679  KU926680 | KU945745  KU945745 | KU945806  KU945807 |
| TE2 | Tenerife, Pal-Mar | KU945686  KU945687 | KU926681  KU926682 | KU945747  KU945748 | KU945808  KU945809 |
| *Iberian Peninsula* | | | | | |
| IB1 | Spain, Cáceres, Santiago de Alcántara | KU945640  KU945641 | KU926635  KU926636 | KU945700  KU945701 | KU945762  KU945763 |
| IB2 | Spain, Almería, Pulpí | KU945642  KU945643 | KU926637  KU926638 | KU945702  KU945703 | KU945764  KU945765 |
| *North-western Africa* | | | | | |
| MO1 | Morocco, Safi Cape | KU945658  KU945659 | KU926653  KU926654 | KU945718  KU945719 | KU945780  KU945781 |
| MO2 | Morocco, Zegangane | KU945664  KU945665 | KU926659  KU926660 | KU945724  KU945725 | KU945786  KU945787 |
| MO3 | Morocco, Hassi-Berkane | KU945666  KU945667 | KU926661  KU926662 | KU945726  KU945727 | KU945788  KU945789 |
| MO4 | Morocco, Had-Rouadi | KU945668  KU945669 | KU926663  KU926664 | KU945728  KU945729 | KU945790  KU945791 |
| MO5 | Morocco, Beni-Sidel | KU945670  KU945671 | KU926665  KU926666 | KU945730  KU945731 | KU945792  KU945793 |
| **Table S1** Continued | | | | | |
| Code | Location | ETS | ITS | *psb*A-*trn*H | *psb*J*-pet*A |
| MO6 | Morocco, Sidi-Bou-Othmane | KU945672  KU945673 | KU926667  KU926668 | KU945732  KU945733 | KU945794  KU945795 |
| MO7 | Morocco, Oued El-Abid Gorges | KU945674  KU945675 | KU926669  KU926670 | KU945734  KU945735 | KU945796  KU945797 |
| MO8 | Morocco, Ouzaghar | KU945676  KU945677 | KU926671  KU926672 | KU945736  KU945737 | KU945798  KU945799 |
| MO9 | Morocco, Oued Assaka | KU945678  KU945679 | KU926673  KU926674 | KU945738  KU945739 | KU945800  KU945801 |
| MO10 | Morocco, Beddouza | KU945660  KU945661 | KU926655  KU926656 | KU945720  KU945721 | KU945782  KU945783 |
| MO11 | Morocco, Safi | KU945662 | KU926657 | KU945722 | KU945784 |
| MO12 | Morocco, Jebel Agouti, Agadir, Melloul | KU945663 | KU926658 | KU945723 | KU945785 |
| *North-eastern Africa and Arabian Peninsula* | | | | | |
| SA1 | Saudi Arabia, Jabal Hada | KU945680 | KU926675 | KU945741 | KU945802 |
| SA2 | Saudi Arabia, Al-Baha | KU945681 | KU926676 | KU945742 | KU945803 |
| SU | Sudan, Arkawit, Jebel Elsit | KU945682  KU945683 | KU926677  KU926678 | KU945743  KU945744 | KU945804  KU945805 |
| SO | Yemen, Socotra Island, Fiheri Park | KU945688 | KU926683 | KU945749 | KU945810 |
| Outgroup | |  |  |  |  |
| *S. megalantha* | | KU945689 | KC692563 | KU945740 | KU945755 |
| -: no accession. | | | | | |

| **Table S2** Taxa used in the ITS data set for dating the origin and diversification of *Scrophularia arguta*, including GenBank accession numbers (GBN). *Scrophularia arguta* samples used in the analysis are the same that in Table S1. | |
| --- | --- |
| Taxa | GBN |
| *Scrophularia atrata* Pennell | HQ130081 |
| *Scrophularia auriculata* L. | KC692527 |
| *Scrophularia buergeriana* Miq. | HQ130070 |
| *Scrophularia bourgeana* Lange in Willk. & Lange | KC692528 |
| *Scrophularia californica* subsp. *floribunda* (Greene) R.J. Shaw | KR361737 |
| *Scrophularia calliantha* Webb & Berthel. | KC692530 |
| *Scrophularia canina* subsp. *bicolor* (Sm.) Greuter | KC692532 |
| *Scrophularia catariifolia* Boiss. & Heldr. | KC692534 |
| *Scrophularia chrysantha* Jaub. & Spach | KC692535 |
| *Scrophularia cinerascens* Boiss. | KR361738 |
| *Scrophularia crassipedunculata* Attar & Joharchi | KC692537 |
| *Scrophularia crassiuscula* Grau | KC692538 |
| *Scrophularia deserti* Delile | KR361739 |
| *Scrophularia desertorum* (Munz) R.J. Shaw | HQ130087 |
| *Scrophularia duplicatoserrata* Makino | HQ130076 |
| *Scrophularia eggersii* Urb. | HQ130090 |
| *Scrophularia eriocalyx* Emb. & Maire | KC692540 |
| *Scrophularia floribunda* Boiss. & Balansa | KC692541 |
| *Scrophularia fontquerii* Ortega Oliv. & Devesa | KC692542 |
| *Scrophularia frutescens* L. | KR361740 |
| *Scrophularia glabrata* Aiton | KR361741 |
| *Scrophularia grandiflora* DC. | KC692545 |
| *Scrophularia grayana* Maxim. ex Kom. | KC692546 |
| *Scrophularia herminii* Hoffmanns. & Link | KR361742 |
| *Scrophularia heterophylla* Willd. | KR361743 |
| *Scrophularia hirta* Lowe | KC692550 |
| *Scrophularia hypericifolia* Wydler | KC692551 |
| *Scrophularia ilwensis* K. Koch | KR361745 |
| *Scrophularia jallui* (Gattef. & Weiller) Ibn Tattou | KC692553 |
| *Scrophularia kakudensis* Franch. | HQ130075 |
| *Scrophularia koraiensis* Nakai | EU165340 |
| *Scrophularia kurdica* subsp. *glabra* Grau | JF409905 |
| *Scrophularia laevigata* var. *pubescens* Maire | KR361746 |
| *Scrophularia laevis* Wooton & Standl. | HQ130077 |
| *Scrophularia lanceolata* Pursh. | KC692555 |
| *Scrophularia laxiflora* Lange | KC692556 |
| *Scrophularia lepidota* Boiss. | KC692557 |
| *Scrophularia libanotica* Boiss. | KC692558 |
| *Scrophularia lucida* L. | KC692560 |
| *Scrophularia lunariifolia* Boiss. & Balansa ex Boiss. | KC692558 |
| *Scrophularia lyrata* Willd. | KR361747 |
| **Table S2** Continued. |  |
| Taxa | GBN |
| *Scrophularia macrantha* Greene ex Stiefelh. | HQ130092 |
| *Scrophularia marilandica* L. | HQ130085 |
| *Scrophularia megalantha* Rech. f. | KC692563 |
| *Scrophularia micrantha* Desv. ex Ham. | HQ130088 |
| *Scrophularia minutiflora* Pennell | HQ130091 |
| *Scrophularia montana* Wooton | HQ130082 |
| *Scrophularia multiflora* Pennell | HQ130078 |
| *Scrophularia musashiensis* Bonati | HQ130073 |
| *Scrophularia* cf. *myriophylla* Boiss. & Heldr. | KC692565 |
| *Scrophularia ningpoensis* Hemsl. | KC692566 |
| *Scrophularia olympica* Boiss. | KC692568 |
| *Scrophularia orientalis* L. | KC692569 |
| *Scrophularia oxyrrhyncha* Coincy | KC692570 |
| *Scrophularia pauciflora* Benth. | KC692571 |
| *Scrophularia peregrina* L. | KU926689 |
| *Scrophularia peyronii* Post | KC692573 |
| *Scrophularia pinardii* Boiss. | KC692574 |
| *Scrophularia racemosa* Lowe | KR361748 |
| *Scrophularia reuteri* Daveau | KC692575 |
| *Scrophularia rosulata* Stiefelh. | KC692577 |
| *Scrophularia sambucifolia* L. | KC692579 |
| *Scrophularia scopolii* Hoppe ex Pers*.* | KR361749 |
| *Scrophularia scorodonia* L. | KC692582 |
| *Scrophularia serrata* Rydb. | HQ130083 |
| *Scrophularia smithii* Hornem. | KC692583 |
| *Scrophularia sublyrata* Brot. | KC692584 |
| *Scrophularia tanacetifolia* Willd. | KC692585 |
| *Scrophularia* cf. *trichopoda* Boiss*.* & Balansa | KC692587 |
| *Scrophularia trifoliata* L. | KC692588 |
| *Scrophularia umbrosa* Dumort. | KR361751 |
| *Scrophularia valdesii* Ortega Oliv. & Devesa | KC692590 |
| *Scrophularia viciosoi* Ortega Oliv. & Devesa | KC692592 |
| *Scrophularia xylorrhiza* Boiss. & Hausskn. ex Boiss. | KC692594 |
| *Scrophularia yoshimurae* T. Yamaz | HQ130072 |
| Outgroup |  |
| *Teedia lucida* Rudolphi | AF375148 |
| *Verbascum nigrum* L. | HQ130064 |
| *Verbascum virgatum* Stokes | KC692522 |
| *S. peregrina*: voucher, UNEX-35912; collectors, M. Navarro, A. Ortega-Olivencia & F.J. Valtueña. | |

| **Table S3** Characteristics of DNA sequence datasets and number of unambiguous indels used in the analysis of *Scrophularia arguta*. | | | | | | |
| --- | --- | --- | --- | --- | --- | --- |
|  | ITS | ETS | nDNA | *psb*A-*trn*H | *psb*J-*pet*A | cpDNA |
| Alignment length (bp) | 581 | 377 | 958 | 447 | 1076 | 1523 |
| Ungappep length sequences | 571-581 | 369-377 | 948-956 | 279-436 | 969-1059 | 1296-1483 |
| Pairwise % identity | 99.2 | 99.4 | 97.3 | 74.5 | 98.0 | 90.7 |
| Variable characters | 37 | 13 | 50 | 38 | 31 | 69 |
| Unambiguous complex indels | - | - | - | 3 | 6 | 9 |

| **Table S4** Bayes factor (BF) support for the significant connections (BF > 3) between geographical areas based on BSSVS analysis of cpDNA in *Scrophularia arguta* by using symmetrical and asymmetrical models. | |
| --- | --- |
| Geographical area connection | BF |
| *Symmetrical model* |  |
| Socotra Island / E Africa | 208.11 |
| E Africa / Iberian Peninsula | 43.50 |
| NW Africa / E Africa | 27.94 |
| Canary Islands / Arabian Peninsula | 7.65 |
| E Africa / Arabian Peninsula | 3.74 |
| *Asymmetrical model* |  |
| E Africa / Socotra Island | 192.54 |
| E Africa / Iberian Peninsula | 60.71 |
| E Africa / NW Africa | 40.95 |
| Arabian Peninsula / Canary Islands | 9.41 |
| E Africa / Arabian Peninsula | 7.54 |
| E Africa / Canary Islands | 4.02 |
| Canary Islands / Arabian Peninsula | 3.89 |

**Fig. S1** BEAST chronogram of *Scrophularia* based on ITS sequence variation. Posterior probabilities of clades are indicated above branches (only PP ≥ 0.90). The 95% posterior density distribution of node ages is shown in the node bars (only branches with a PP ≥ 0.90). The scale is in million years. Arrows indicate calibration points used in the analyses (A, 26.77 ± 4.27 Ma; B, 15.92 ± 3.29 Ma; C, 10.20 ± 2.36 Ma).

**
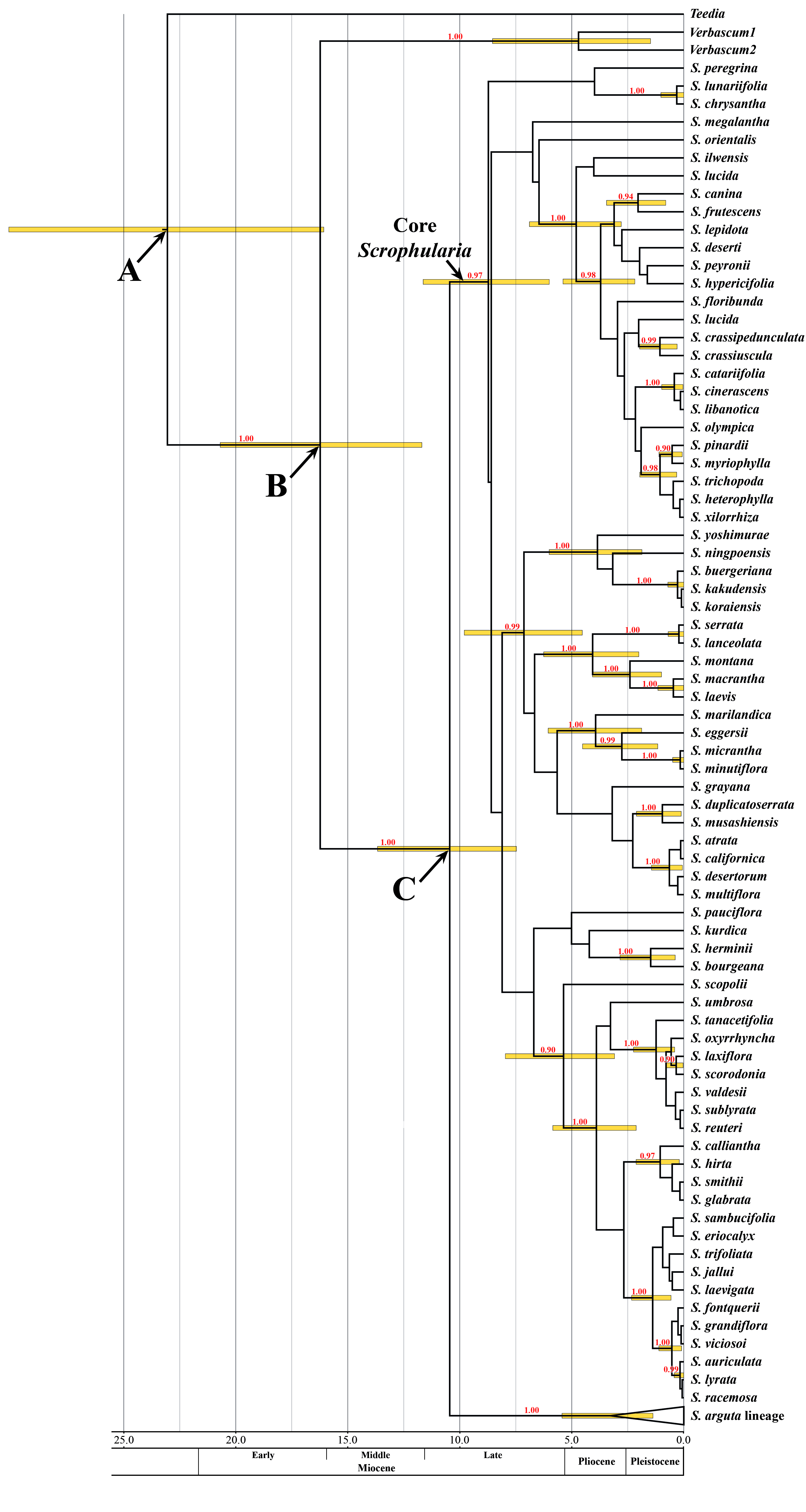
**

**Fig. S2** BEAST chronogram of *Scrophularia arguta* based on two cpDNA sequences (*psb*A*-trn*H*/psb*J*-pet*A). Values above branches are posterior probability values (PP) and under branches are maximum likelihood (ML) bootstrap (BS) values. Only PP ≥ 0.90 and ML BS ≥ 65 are shown. Grey background indicates the Canarian populations. Clades as in Fig. 3 are indicated on the right side.

**
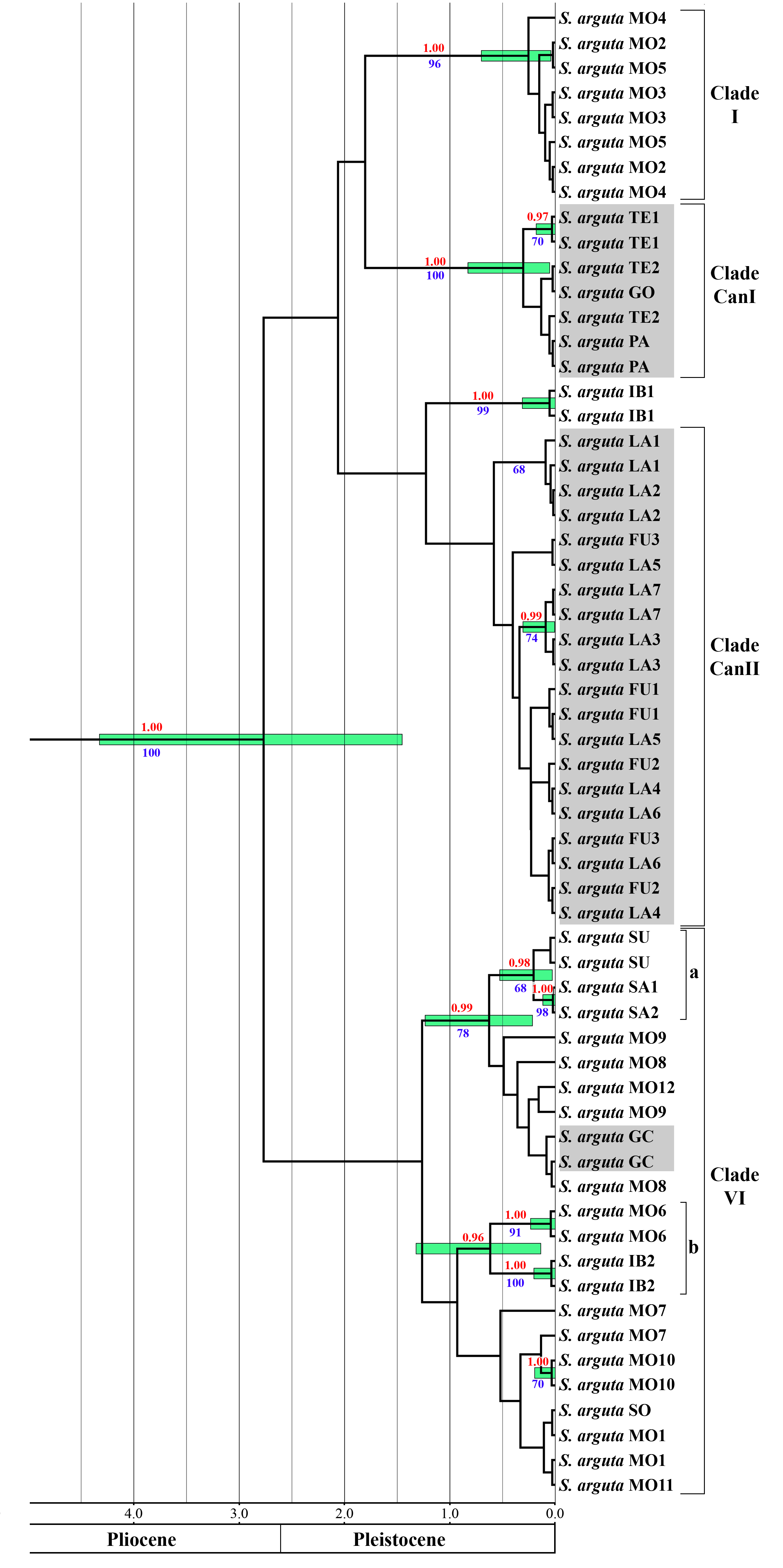
**

**Fig. S3** BEAST chronogram of *Scrophularia arguta* based on two nDNA sequences (ITS/ETS). Values above branches are posterior probability values (PP) and under branches are maximum likelihood (ML) bootstrap (BS) values. Only PP ≥ 0.90 and ML BS ≥ 65 are shown. Grey background indicates the Canarian populations. Clades as in Fig.2 and Fig. 3 are indicated on the right side.


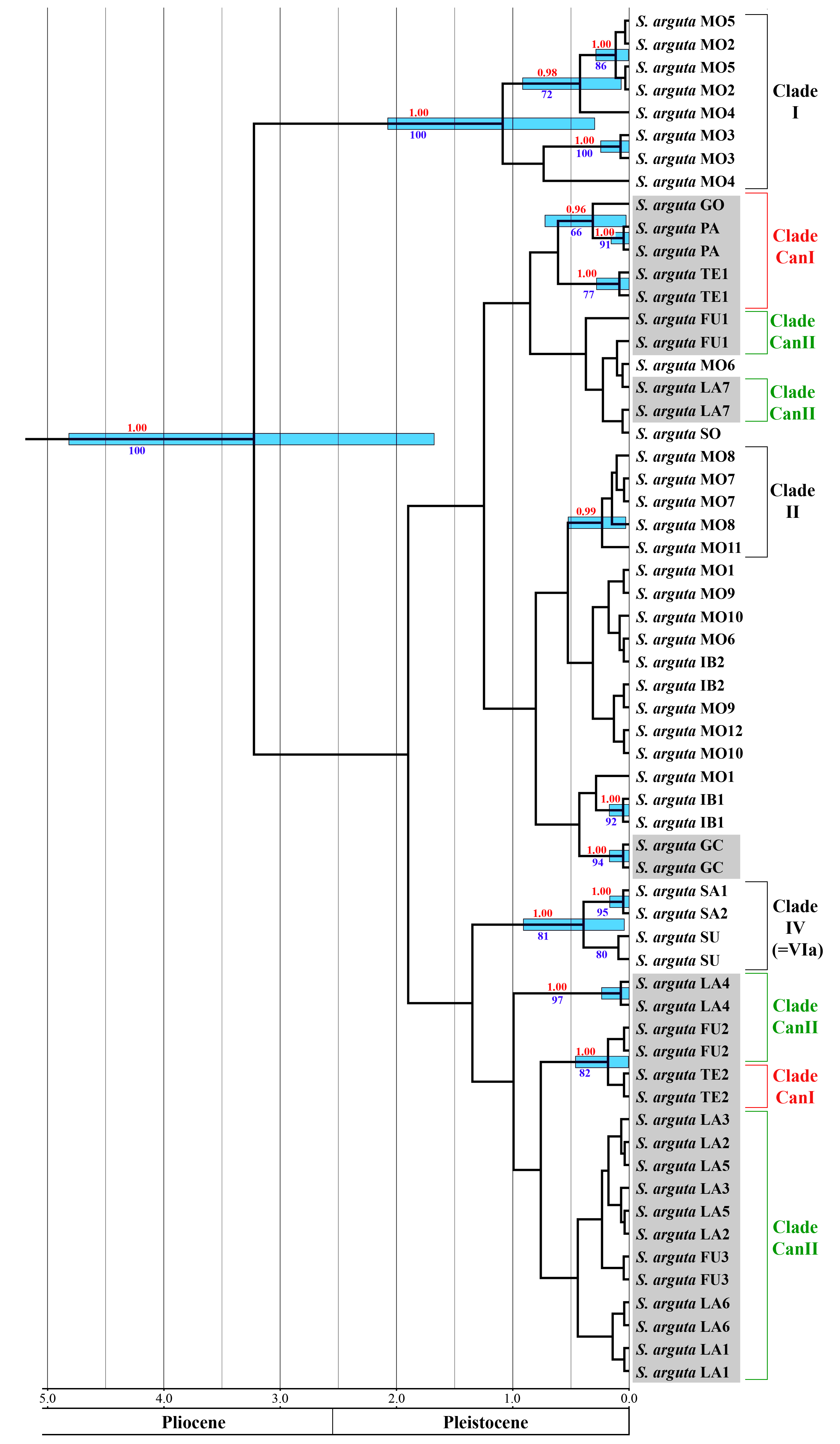


**Fig. S4** Maximum clade credibility tree generated by BSSVS analysis of cpDNA in *Scrophularia arguta* considering symmetrical (a) and asymmetrical (b) models. Branches are colored according to highest probability inferred ancestral geographical range. Highest probability of geographical range is indicated above branches (only values < 1.00).


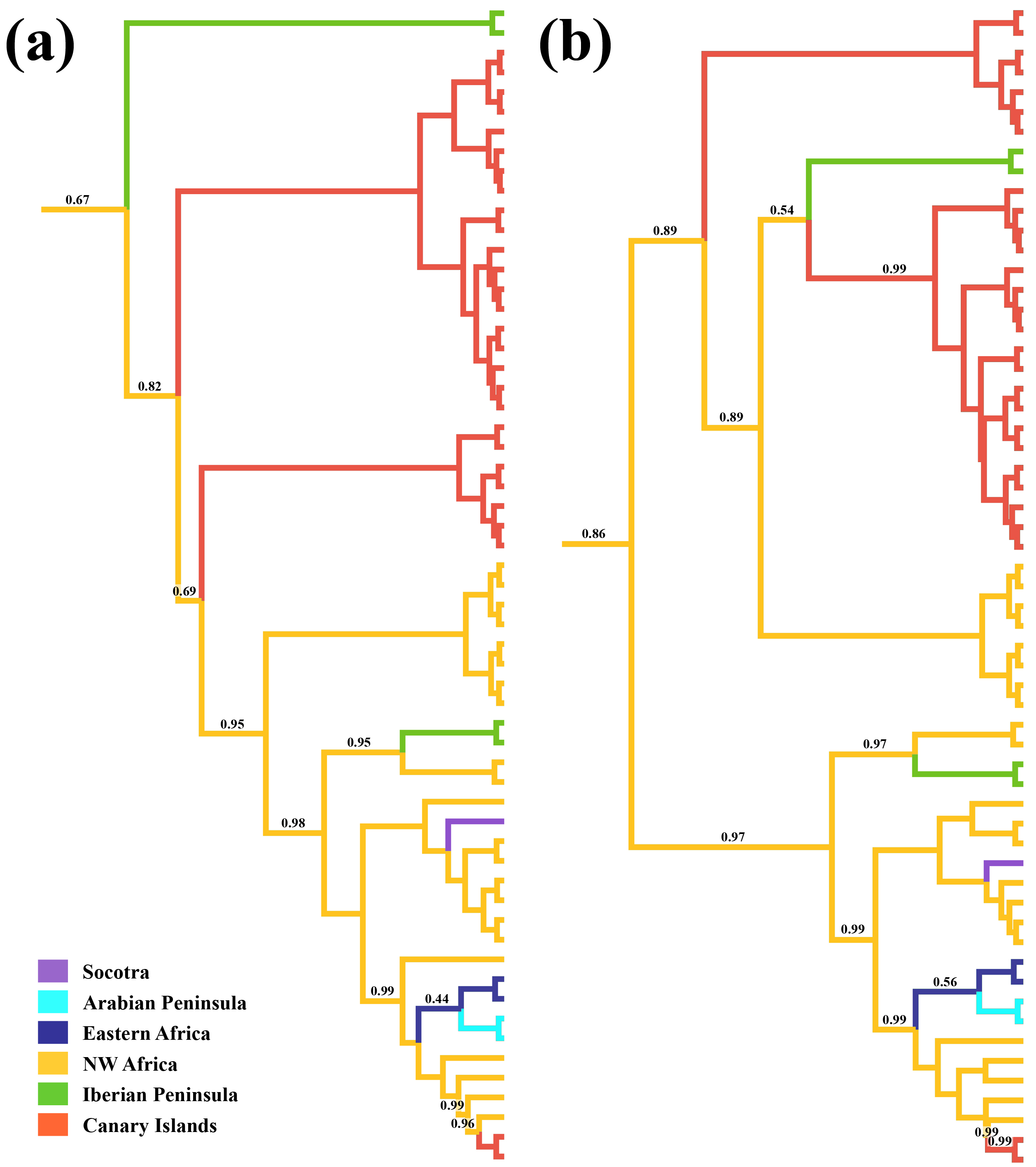

Supplement: Supplementary file 1 — Table S1. Material of Scrophularia arguta and outgroup taxon (S. megalantha) studied, including population code (as in Table 1), location and GenBank accession numbers for the DNA sequences analyzed. Table S2. Taxa used in the ITS data set for dating the origin and diversification of Scrophularia arguta, including GenBank accession numbers (GBN). Table S3. Characteristics of DNA sequence datasets and number of unambiguous indels used in the analysis of Scrophularia arguta. Table S4. Bayes factor (BF) support for the significant connections (BF > 3) between geographical areas based on BSSVS analysis of cpDNA in Scrophularia arguta by using symmetrical and asymmetrical models. Figure S1. BEAST chronogram of Scrophularia based on ITS sequence variation. Posterior probabilities of clades are indicated above branches (only PP ≥ 0.90). Figure S2. BEAST chronogram of Scrophularia arguta based on two cpDNA sequences (psbA‐trnH/psbJ‐petA). Figure S3. BEAST chronogram of Scrophularia arguta based on two nDNA sequences (ITS/ETS). Values above branches are posterior probability values (PP) and under branches are maximum‐likelihood (ML) bootstrap (BS) values. Figure S4. Maximum clade credibility tree generated by BSSVS analysis of cpDNA in Scrophularia arguta considering symmetrical (a) and asymmetrical (b) models. Branches are colored according to highest probability inferred ancestral geographical range. [file ECE3-6-4258-s001.docx]
